# RETRACTION: AKIP1 Expression in Tumor Tissue as a New Biomarker for Disease Monitoring and Prognosis in Non‐Small Cell Lung Cancer: Results of a Retrospective Study

**DOI:** 10.1002/jcla.70239

**Published:** 2026-04-13

## Abstract

**RETRACTION**: Y. Liu, J. Tian, D. Qin, J. Liu, Y. Xie, “AKIP1 Expression in Tumor Tissue as a New Biomarker for Disease Monitoring and Prognosis in Non‐Small Cell Lung Cancer: Results of a Retrospective Study,” *Journal of Clinical and Laboratory Analysis* 34, no. 4 (2019): e23128, https://doi.org/10.1002/jcla.23128.

The above article, published online on 29 November 2019 in Wiley Online Library (wileyonlinelibrary.com), has been retracted by agreement between the journal Editor‐in‐Chief, Rong Fu; and Wiley Periodicals, LLC. The retraction has been agreed due to concerns raised by a third party, which identified compromised data and systematic issues in the published article, specifically, inappropriate image duplications in Figure 2 (panel (A)) that had been previously published by a different author group. In addition, Figures 3 and 4 were found to show unattributed similarities to figures in another article published around the same time by a different author group. The authors and their institutes were unresponsive when these concerns were brought to their attention. Accordingly, the article is retracted as the editors have lost trust in the reliability of the data and the results presented.

**RETRACTION**: Y. Liu, J. Tian, D. Qin, J. Liu, Y. Xie, “AKIP1 Expression in Tumor Tissue as a New Biomarker for Disease Monitoring and Prognosis in Non‐Small Cell Lung Cancer: Results of a Retrospective Study,” *Journal of Clinical and Laboratory Analysis* 34, no. 4 (2019): e23128, https://doi.org/10.1002/jcla.23128.

The above article, published online on 29 November 2019 in Wiley Online Library (wileyonlinelibrary.com), has been retracted by agreement between the journal Editor‐in‐Chief, Rong Fu; and Wiley Periodicals, LLC. The retraction has been agreed due to concerns raised by a third party, which identified compromised data and systematic issues in the published article, specifically, inappropriate image duplications in Figure 2 (panel (A)) that had been previously published by a different author group. In addition, Figures 3 and 4 were found to show unattributed similarities to figures in another article published around the same time by a different author group. The authors and their institutes were unresponsive when these concerns were brought to their attention. Accordingly, the article is retracted as the editors have lost trust in the reliability of the data and the results presented.

